# The association of depression and posttraumatic stress disorder with the metabolic syndrome in a multi-ethnic cohort: the HELIUS study

**DOI:** 10.1007/s00127-018-1533-y

**Published:** 2018-05-23

**Authors:** Marieke J. van Leijden, Brenda W. J. H. Penninx, Charles Agyemang, Miranda Olff, Marcel C. Adriaanse, Marieke B. Snijder

**Affiliations:** 1 Department of Public Health, Academic Medical Center, Meibergdreef 15, 1105 AZ Amsterdam, The Netherlands; 2 Department of Psychiatry, Amsterdam Public Health Research Institute, VU University Medical Center, Oldenaller 1, 1081 HL Amsterdam, The Netherlands; 3 Department of Psychiatry, Academic Medical Center, Meibergdreef 5, 1105 AZ Amsterdam, The Netherlands; 4 Department of Health Sciences, Vrije Universiteit Amsterdam, De Boelelaan 1085, 1081 HV Amsterdam, The Netherlands

**Keywords:** Metabolic syndrome, Depression, Posttraumatic stress disorder, Ethnicity, HELIUS study

## Abstract

**Purpose:**

Depression and posttraumatic stress disorder (PTSD) may be linked to the metabolic syndrome (MetS). Consistency of this association across ethnic groups and the influence of comorbidity of depression/PTSD were examined.

**Methods:**

Cross-sectional baseline data from the HELIUS study were used (4527 Dutch, 2999 South-Asian Surinamese, 4058 African Surinamese, 2251 Ghanaian, 3522 Turkish and 3825 Moroccan participants). The Patient Health Questionnaire-9 (PHQ-9) (score range 0–27) measured depressive symptoms. A 9-item questionnaire (score range 0–9) measured PTSD symptoms. The MetS was defined according to the International Diabetes Federation. The association of a depressed mood (PHQ-9 sum score ≥ 10) and severe PTSD symptoms (sum score ≥ 7) with the MetS was examined using logistic regression. Interaction with ethnicity and between a depressed mood and severe PTSD symptoms was tested.

**Results:**

A depressed mood was associated with the MetS [OR (95% CI) = 1.37 (1.24–1.51)] in the total sample and consistent across ethnic groups (*p* values for interaction all > 0.05). Severe PTSD symptoms were significantly associated with the MetS in the Dutch [OR (95% CI) = 1.71 (1.07–2.73)]. The South-Asian Surinamese, Turks and Moroccans showed weaker associations than the Dutch (*p* values for interaction all < 0.05). A depressed mood and severe PTSD symptoms did not interact in the association with the MetS (*p* values for interaction > 0.05).

**Conclusions:**

A depressed mood was consistently associated with the MetS across ethnic groups, but the association between severe PTSD symptoms and the MetS maybe ethnicity dependent. The association with the MetS was not different in case of depressed mood/severe PTSD symptoms comorbidity.

**Electronic supplementary material:**

The online version of this article (10.1007/s00127-018-1533-y) contains supplementary material, which is available to authorized users.

## Introduction

The metabolic syndrome (MetS) is a common cluster of somatic symptoms, including elevated waist circumference, elevated blood pressure, dyslipidemia (reduced HDL and elevated triglyceride levels) and elevated fasting glucose [1]. The MetS contributes to the rising prevalence of cardiovascular disease and type 2 diabetes [2]. Psychopathology may be a risk factor for the MetS; two frequently comorbid mental disorders, depression and posttraumatic stress disorder (PTSD), have been linked to the MetS [3, 4]. The pathways that link depression and PTSD with the MetS are not completely understood, but evidence points towards unhealthy lifestyle, biological stress dysregulation and/or inflammation [5–9].

The association between depression and the MetS has been established; several large meta-analyses show this association [4, 10, 11]. While previous studies mostly used cross-sectional data [4], several large prospective studies have demonstrated an association between depression and subsequent MetS development [11, 12]. Moreover, this association has been examined in diverse populations and in several countries [4]. However, the consistency of this association across ethnic groups remains unclear. One study in the US found an association between depression and the MetS in white Americans, but not in African-Americans [13], while another study found no interaction by ethnicity in a sample including white, Mexican Americans and African-Americans [14]. Similarly, one study found no interaction by ethnicity in the association with metabolic risk factors (BMI, obesity rate, waist-hip ratio, LDL level, and triglyceride level) [15]. In contrast, studies on depression and cardiovascular mortality and coronary heart disease showed an association only in African-Americans, but not in white Americans [16–18]. However, these studies were all conducted in the US and it is unclear how these findings translate to ethnic groups with different migration backgrounds and living conditions.

The association between PTSD and the MetS is poorly established compared to the association between depression and the MetS. In a meta-analysis including 9 cross-sectional studies, Rosenbaum et al. found a 1.82 times higher risk of the MetS for persons with PTSD compared to age- and sex-matched controls [3]. Recently, a study showed a longitudinal association between PTSD and the MetS, controlling for age, sex, ethnicity and educational level [19]. Evidence on this association is still lacking, however. For example, the association between PTSD and the MetS has rarely been studied in large samples [19]. Moreover, the majority of studies on PTSD and the MetS were conducted in veterans [19–25]. Furthermore, similar to depression, there may be ethnic differences in the relationship between PTSD and the MetS. In fact, in one study on the relationship between PTSD and chronic illnesses, the association with diabetes and heart disease was strongest in African-Americans [26]. In contrast, another small study showed an association between PTSD and metabolic risk factors in white American women, but not in African American women [15]. Both studies were conducted in the US. Again, uncertainty remains whether these differences between US ethnic groups are applicable to the European context, and how this translates to the association with the MetS.

As these studies illustrate, there may be differential associations between psychopathology and somatic outcomes between ethnic groups. We know that some ethnic minority groups have an increased risk of developing depression, PTSD and the MetS [27–29]. One could speculate why ethnicity might moderate the association between psychopathology and the MetS. For example, it is possible that some ethnic groups are more prone towards developing the MetS due to a number of risk factors other than depression and PTSD, such as migration-related stressors, perceived ethnic discrimination, biological disposition, unhealthy lifestyle, poor social support and/or socioeconomic status [30–33]. On the other hand, it has been argued that ethnic minorities may be more vulnerable to the effects of psychopathology on somatic health, because these groups may experience less adequate care for psychopathology, which can prolong its course and increase the risk of harmful effects [26]. As the Western society is becoming increasingly multicultural [34], multi-ethnic comparisons of the link between psychopathology and MetS are important to better understand the nature of this comorbidity and its clinical implications in a multicultural society.

Because depression and PTSD are frequently comorbid [35, 36], and may both increase the risk of developing the MetS [3, 4], it is important to investigate both disorders in relation to the MetS. Depression and PTSD have an overlap in symptoms, in particular negative affect and dysphoric arousal [37]. Depression is seen as a confounder in the association between PTSD and the MetS [24, 38]. Additionally, it has been suggested that comorbidity of depression and PTSD increases the risk of the MetS more than PTSD alone [25, 39]. There is, however, a scarcity of studies that investigate the independence of depression and PTSD in their association with the MetS, and the existence of a potential interaction between these disorders in their association with the MetS. It seems plausible that when the two disorders co-occur, the impact on health may be strengthened. After all, previous studies have shown that comorbidity in mental health conditions, due to higher symptom burden or more diverse pathophysiology, have a negative impact on physical health [40, 41]. Consequently, there may be a significant interaction between depression and PTSD in the association with the MetS.

Therefore, the aim in this study was to examine the associations of both symptoms of depression and PTSD with the MetS. We investigated the consistency of these associations across ethnic groups living in the Netherlands and the potential interaction between depression and PTSD in the association with the MetS.

## Methods

### The HELIUS study

The HEalthy LIfe in an Urban Setting (HELIUS) study is a multi-ethnic cohort study aiming to unravel the mechanisms underlying the impact of ethnicity on communicable and non-communicable diseases [42]. Full details on this study are described elsewhere [42]. In brief, baseline data collection took place in 2011–2015 and included people aged 18–70 years from different ethnic groups living in Amsterdam, i.e., those of Dutch, Surinamese, Ghanaian, Moroccan and Turkish origin. Participants were randomly sampled from the municipal register, stratified by ethnicity. Data were collected by a questionnaire and a physical examination in which biological samples were also obtained.

For the current study, we used baseline data of all participants in whom questionnaire data as well as data from the physical examination were available (*n* = 22,165). Javanese Surinamese participants (*n* = 233) and those with an unknown/other Surinamese origin (*n* = 267) or unknown/other ethnic origin (*n* = 48) were excluded because of their relatively small sample sizes. We further excluded participants with missing values on a depressed mood (*n* = 240), severe PTSD symptoms (*n* = 240) and/or on the MetS (*n* = 123). In the current analysis, the total sample consisted of 21,182 participants; 4527 Dutch, 2999 South-Asian Surinamese, 4058 African Surinamese, 2251 Ghanaian, 3522 Turkish and 3825 Moroccan origin participants.

### Ethnicity

Ethnicity was defined according to the country of birth of the participant as well as that of his/her parents, which is currently the most widely accepted assessment of ethnicity in Netherlands [43]. Participants were considered to be of Dutch origin if the person and both parents were born in the Netherlands. Participants were considered to be of non-Dutch origin if the person fulfills either of the following criteria: (1) he or she was born abroad and has at least one parent born abroad (first generation) or (2) he or she was born in the Netherlands but both his/her parents were born abroad (second generation). Surinamese subgroups (i.e., African and South-Asian) were classified according to self-reported ethnic origin.

### Depressive symptoms

Depressive symptoms were assessed using the Patient Health Questionnaire-9 (PHQ-9) [44]. The PHQ-9 determines the prevalence of depressive symptoms over the preceding 2 weeks. Its cross-cultural validity has been demonstrated across the ethnic groups included in the HELIUS study [45]. The PHQ-9 consists of nine items, with a response scale varying from never (0) to nearly every day (3). Cronbach’s alpha was 0.89 in the whole sample and ranged from 0.84 to 0.90 between the ethnic groups. If one of the items was missing, the mean score of the other eight items was used to replace the missing item. If more than one item was missing, the variable was considered missing. Participants having a sum score of 10 or higher were considered to have a ‘depressed mood’ [44].

### PTSD symptoms

PTSD symptoms were measured by a questionnaire. Questions were derived from the PTSD Symptom Scale-Self-Report Version (PSS-SR), an instrument with good validity and high reliability [46]. The questionnaire consisted of nine items: three re-experiencing items, two avoidance items and four hyperarousal items. Cronbach’s alpha was 0.90 in the whole sample and ranged from 0.85 to 0.92 between the six ethnic groups. Answer categories were yes (1) and no (0). We calculated the sum score of the nine PTSD items (ranging from 0 to 9). In case of more than two missing items, the sum score was not calculated and considered as missing. Based on the non-linear association of the PTSD sum score with our main outcome variable (the MetS) (see online resource Table S1), the PTSD sum score variable was dichotomized: participants with a sum score of seven or higher were considered to have ‘severe PTSD symptoms’.

### Metabolic syndrome

The MetS was determined according to the harmonized definition proposed by the International Diabetes Federation (IDF) [1]. By this definition, the MetS is present if at least three of the following five criteria are met (yes/no): (a) elevated fasting glucose (≥ 5.6 mM, or glucose-lowering medication); (b) elevated blood pressure (systolic ≥ 130 and/or diastolic ≥ 85 mm Hg, or blood pressure–lowering medication); (c) reduced HDL-C (< 1.0 mM for men, 1.3 mM for women, or lipid-lowering medication); (d) elevated triglycerides (≥ 1.7 mM, or lipid-lowering medication); and (e) elevated waist circumference (ethnic specific cutoff values were used; for all women ≥ 80 cm, South-Asian men ≥ 90 cm and other men ≥ 94 cm) [1].

### Biomedical measurements

Overnight fasting blood samples were drawn and plasma samples were used to determine the concentration of glucose by spectrophotometry, using hexokinase as primary enzyme (Roche Diagnostics, Tokyo, Japan). Triglycerides and HDL-C were determined by colorimetric spectrophotometry (Roche Diagnostics). Blood pressure was measured in a seated position using a semiautomatic sphygmomanometer (Microlife WatchBP Home; Microlife AG, Widnau, Switzerland). Using appropriate cuff sizes, two readings were taken on the upper left arm at heart level after being seated for at least 5 min. The mean of the two readings was used for analysis. Waist circumference was measured using a non-elastic, flexible tape measure at the level midway between the lower rib margin and the iliac crest. Waist circumference was measured in duplicate, and a third measurement was taken if the difference between the first two measurements was > 1 cm. In addition, all participants were asked to bring their prescribed medications to the research location, which were identified and categorized using the Anatomical Therapeutic Chemical (ATC) classification system [47].

### Covariates

Educational level was used as an indicator of socioeconomic status. It was defined as the highest qualification obtained in the Netherlands or in the country of origin. The categories were: (1) no education or elementary education only, (2) lower vocational or general secondary education, (3) intermediate vocational or higher secondary education and (4) higher vocational education or university. Current smoking (yes/no) and heavy alcohol use (yes/no) were determined by questionnaire. Heavy alcohol use was defined as consuming more than two alcoholic beverages on more than two occasions per week. Physical activity (PA) was self-reported using the Short Questionnaire to Assess Health-Enhancing Physical Activity (SQUASH) questionnaire [48]. Adequate PA (yes/no) is defined as reaching the international goal for PA (moderate- to high-intensity PA for at least 30 min per day on at least five days per week).

### Statistical analyses

Descriptive statistics were used to calculate percentages and means with standard deviations for demographic variables, main variables and covariates. The association of a depressed mood and severe PTSD symptoms with the MetS was investigated using logistic regression analyses. The independent variables were a depressed mood (PHQ-9 sum score ≥ 10) or severe PTSD symptoms (sum score ≥ 7), and the dependent variable was the MetS. We adjusted for confounding by sex, age, ethnicity and educational level. To account for comorbidity of a depressed mood and severe PTSD symptoms, both variables were added to the final model. The correlation between the continuous PHQ-9 sum score and PTSD sum score was poor (Spearman’s rho = 0.44, *p* < 0.01), thus ruling out multicollinearity. Additionally, we tested for effect modification by ethnicity by adding interaction terms to the regression models (using the Dutch as the reference population). Additionally, interaction analyses were performed to examine whether a depressed mood and severe PTSD symptoms interact in the association with MetS. Finally, we adjusted for lifestyle factors (current smoking, heavy alcohol use and compliance to PA norm) to study whether these variables attenuate the associations of a depressed mood and severe PTSD symptoms with the MetS. Because some studies report sex- or age-specific associations between depression and the MetS [49], we also tested for effect-modification by sex and age groups by adding interaction terms to the regression models. However, no significant effect modification by age or sex was found in our study (data not shown). IBM SPSS Statistics version 24.0 was used for statistical analysis. *p* values of < 0.05 were deemed statistically significant.

## Results

### Sample characteristics

Table 1 shows the characteristics of the study population, for the total sample (*n* = 21,182) and stratified by ethnicity. In all ethnic groups, the percentage of women was larger than men. The mean age was 44.2 (SD = 13.2) years, but the Turkish and Moroccan groups were slightly younger than the other ethnic groups. The Dutch were most often highly educated. The PHQ-9 and PTSD sum scores show the highest mean scores among the South-Asian Surinamese, Turkish and Moroccan groups. The smoking rate was remarkably low among Ghanaians. The Dutch are heavy alcohol users most often, and are most often compliant to the PA norm.

**Table 1:** Characteristics of the study population, in the total sample and by ethnicity

	Total sample (*n* = 21,182)	Dutch (*n* = 4527)	South-Asian Surinamese (*n* = 2999)	African Surinamese (*n* = 4058)	Ghanaian (*n* = 2251)	Turkish (*n* = 3522)	Moroccan (*n* = 3825)
Female (%)	57.7	54.1	54.6	61.0	61.2	54.9	61.2
Age in years, mean (SD)	44.21 (13.22)	46.18 (14.04)	45.44 (13.41)	47.87 (12.55)	44.64 (11.21)	40.27 (12.16)	40.41 (12.93)
Education (%)^a^							
1 (lowest)	17.6	3.3	14.2	5.5	28.6	31.3	31.0
2	26.2	14.2	33.3	35.7	40.0	25.0	17.8
3	29.2	21.9	29.3	35.8	25.1	28.7	33.6
4 (highest)	27.0	60.7	23.2	23.1	6.2	15.0	17.6
PHQ-9 sum score, Mean (SD)	4.76 (5.20)	3.57 (3.75)	5.41 (5.84)	3.92 (4.61)	3.37 (4.31)	6.42 (6.01)	5.86 (5.68)
PTSD sum score, Mean (SD)	1.40 (2.40)	0.90 (1.79)	1.72 (2.64)	1.22 (2.20)	1.03 (1.99)	1.92 (2.79)	1.67 (2.69)
Lifestyle factors (%)							
Current smoking	24.0	24.7	28.4	31.6	4.4	34.6	13.4
Heavy alcohol use	11.3	33.4	8.5	9.2	3.8	3.2	1.5
Compliant to PA norm	56.7	75.7	53.4	61.4	53.4	42.1	47.0
MetS components (%)							
Reduced HDL-C	31.6	19.6	46.0	26.9	20.1	42.4	36.0
Elevated triglycerides	19.3	17.2	32.0	14.6	10.4	26.5	15.3
Elevated fasting glucose	30.5	25.5	41.9	30.4	28.5	28.5	30.7
Elevated blood pressure	46.5	38.8	51.6	60.4	66.7	38.6	32.1
Elevated waist circumference	64.9	53.6	70.1	65.2	69.1	69.6	67.1

^a^(1) no education or elementary education only, (2) lower vocational or general secondary education, (3) intermediate vocational or higher secondary education and (4) higher vocational education or university

**Fig. 1:**
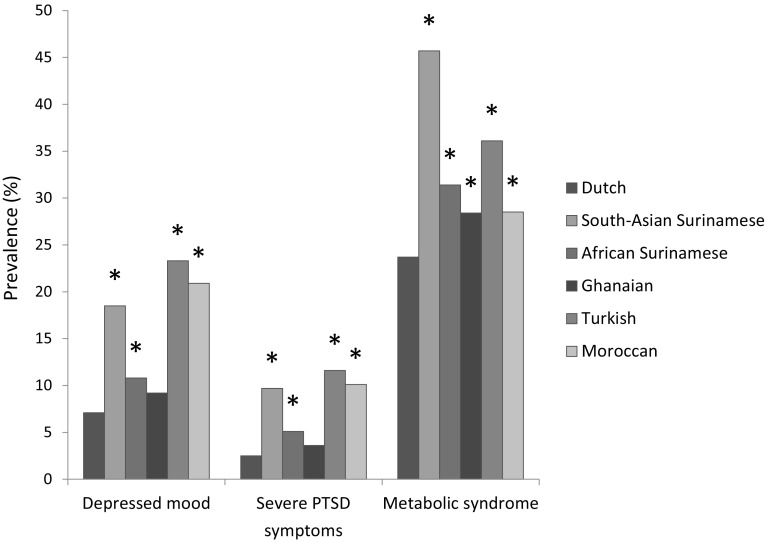
Prevalence of depressed mood (PHQ-9 sum score ≥ 10), severe PTSD symptoms (PTSD sum score ≥ 7) and the MetS, by ethnicity. Asterisk: Prevalence is significantly higher than in the Dutch, after adjustment for age, sex and educational status (*p* value < 0.05)

Figure 1 shows the prevalence of the main variables: depressed mood, severe PTSD symptoms and the MetS. Turks, Moroccans and South-Asian Surinamese had the highest prevalence of a depressed mood, followed by the African Surinamese and Ghanaians. A depressed mood was lowest in the Dutch. Severe PTSD symptoms followed the same pattern, with the Turks having the highest prevalence, and the Dutch having the lowest. The MetS was most common in the South-Asian Surinamese, followed by the Turks, the African Surinamese, then the Moroccans and Ghanaians and finally the Dutch. When looking at the individual components of the MetS (Table 1), elevated fasting glucose was mostly seen in the South-Asian Surinamese, while elevated blood pressure mostly presented itself in the Ghanaians and African Surinamese. South-Asian Surinamese and the Turks had the highest prevalence of dyslipidemia.

### Main analyses

Table 2 shows the association between a depressed mood and the MetS in the total sample and by ethnicity. This association was consistent across ethnic groups, after adjustment for age and sex (model 1) and additional adjustment for educational level (model 2). The association between a depressed mood and the MetS did not reach statistical significance in the Ghanaians, but it was not significantly different from the association in the Dutch population when tested (p-value interaction 0.18). Adjusting for severe PTSD symptoms attenuated these results slightly (model 3). After adjusting for current smoking, heavy alcohol use and compliance to the PA norm, the association did not change [adjusted odds ratio (95% confidence interval) = 1.36 (1.23–1.50) in the total sample]. When taking the continuous PHQ-9 scale as a determinant, instead of the dichotomous depressed mood variable, similar positive associations consistent across ethnic groups were shown (see online resource table S2).

**Table 2:** Association (ORs with 95% CI) of a depressed mood (PHQ-9 sum score ≥ 10) and severe PTSD symptoms (PTSD sum score ≥ 7) with the MetS, in the total sample and by ethnicity

	Total sample (*n* = 21,182)	Dutch (*n* = 4527)	South-Asian Surinamese (*n* = 2999)	African Surinamese (*n* = 4058)	Ghanaian (*n* = 2251)	Turkish (*n* = 3522)	Moroccan (*n* = 3825)
Depressed mood							
Model 1	**1.24 (1.13–1.36)**	**1.80 (1.35–2.40)**	**1.48 (1.20–1.82)**	**1.73 (1.38–2.18)**	1.13 (0.81–1.57)	1.28 (1.07–1.54)*	**1.26 (1.04–1.53)**
Model 2	**1.17 (1.06–1.28)**	**1.52 (1.14–2.04)**	**1.43 (1.15–1.76)**	**1.67 (1.33–2.10)**	1.12 (0.80–1.55)	**1.22 (1.02–1.46)**	**1.24 (1.03–1.51)**
Model 3	**1.37 (1.24–1.51)**	**1.37 (1.01–1.87)**	**1.57 (1.24–1.98)**	**1.64 (1.29–2.10)**	1.09 (0.77–1.54)	**1.22 (1.01–1.49)**	**1.32 (1.07–1.63)**
Severe PTSD symptoms							
Model 1	**1.16 (1.02–1.31)**	**2.33 (1.51–3.61)**	0.98 (0.75–1.29)*	**1.43 (1.04–1.96)**	1.23 (0.73–2.05)	1.12 (0.89–1.42)*	0.96 (0.74–1.24)*
Model 2	1.11 (0.98–1.25)	**1.97 (1.26–3.09)**	0.95 (0.72–1.25)*	**1.37 (1.00-1.88)**	1.20 (0.72–2.01)	1.09 (0.86–1.38)*	0.94 (0.72–1.21)*
Model 3	0.94 (0.82–1.08)	**1.71 (1.07–2.73)**	0.74 (0.55–1.01)*	1.07 (0.76–1.50)	1.15 (0.67–1.98)	0.98 (0.76–1.27)*	0.81 (0.61–1.07)*

Model 1: adjusted for age and sex (and for ethnicity in the total sample only)

Model 2: adjusted for age, sex and educational level (and for ethnicity in the total sample only)

Model 3: adjusted for age, sex, educational level and comorbid depressed mood or severe PTSD symptoms (and for ethnicity in the total sample only)

*p* value < 0.05 are in bold

*Association is significantly different compared to the association in the Dutch population (*p* value interaction < 0.05)

Table 2 also shows the association between severe PTSD symptoms and the MetS in the total sample and by ethnicity. The South-Asian Surinamese, Turks and Moroccans showed weaker associations compared to the Dutch population (*p* values for interaction < 0.01; *p* = 0.01; *p* < 0.01, respectively). In the Dutch and the African Surinamese only, severe PTSD symptoms were significantly associated with the MetS after adjustment for age, sex and educational level (models 1 and 2). This association was attenuated by additional adjustment for a depressed mood, and only remained significant among the Dutch (model 3). After additional adjustment for current smoking, heavy alcohol use and compliance to the PA norm, the association did not change [adjusted odds ratio (95% confidence interval) = 1.73 (1.07–2.78) among the Dutch].

### Interaction between a depressed mood and severe PTSD symptoms

A depressed mood and severe PTSD symptoms did not have a significant interaction in the association with the MetS, neither in the total sample nor in the individual ethnic groups (see online resource table S3).

## Discussion

This study is unique in that it examines the association of both symptoms of depression and PTSD with the MetS across large samples of six diverse ethnic groups living in Amsterdam, the Netherlands. Participants with a depressed mood had increased odds of the MetS, consistent across ethnic groups. Participants with severe PTSD symptoms had increased odds of the MetS in the Dutch host population, but this association was not observed in the ethnic minority groups. Comorbidity of a depressed mood and severe PTSD symptoms did not moderate the association between the two mental disorders and the MetS.

This study extends the evidence for the association between depressive symptoms and the MetS [4], by demonstrating consistency of this association across ethnic groups. While some previous studies in the US suggest that ethnic minorities (African-Americans) have stronger associations between depression and cardiovascular disease [13, 16–18, 50], this was not observed in the association with the MetS in our population. This discrepancy may be caused by the differences in migration background and living conditions between ethnic groups in the US and Europe [30].

This study adds to previous studies on the association between PTSD and the MetS, by investigating this association in a diverse population, in terms of sex and ethnicity. Evidence derived from previous studies, with populations consisting mostly of male, white American veterans [19–25], suggests a strong association between PTSD and the MetS, independent of depression [19]. However, we only observed an association in the Dutch host population, but not in the ethnic minority groups. This is in line with a previous study that showed an association between PTSD and metabolic risk factors (BMI, obesity rates, abdominal obesity and triglycerides) in white Americans, but not in African-Americans [15]. The results from this study lead us to suggest that in ethnic minority groups, the risk of developing the MetS may be more strongly influenced by other risk factors, such as depressive symptoms, disadvantageous biological factors that originate in early life [51] or ethnicity-specific dietary patterns [52] andexercise beliefs [53].

Because we investigated both a depressed mood and severe PTSD symptoms in the association with the MetS, we were able to test the interaction between these mental disorders. We found no evidence that the association between a depressed mood and severe PTSD symptoms and the MetS is moderated by comorbidity of the two disorders. This finding is at odds with a small previous study that reported an increased risk of the MetS when PTSD is comorbid with depression [25].This study, however, did not use statistical analysis to test this interaction. Another study examining PTSD symptoms and onset of cardiovascular events did not find an interaction with depression [54]. In contrast, a study examining the association between PTSD and diabetes among asylum seekers in the Netherlands, found an interaction with comorbid depression: they only found an association between PTSD and diabetes in the group that was not depressed [55].

This study has several strengths. This study is the first European study to describe both depression and PTSD symptomatology in relation to the MetS among several ethnic groups, including large sample sizes and detailed information on psychological, biological and anthropometric measurements. With over 20,000 participants, this study is well powered, even after stratifying by ethnicity. Nevertheless, the results of this study need to be interpreted with caution given certain limitations. First, the PTSD questionnaire is not yet validated. While the questions were derived from another validated questionnaire [46], the (transcultural) validity of this selection of questions is unclear and should be investigated. Second, a limitation of this study is its cross-sectional nature, which implies we should be careful making causal inferences. There may be a bidirectional association between depression and the MetS [11]. However, a large longitudinal study showed depressive symptoms predicting MetS development, but MetS components did not predict depressive symptoms [12]. Similarly, a large longitudinal study showed that PTSD symptoms predict subsequent MetS development, but the MetS did not predict development of PTSD symptoms [19]. The lack of an association between the MetS and PTSD development is also shown in another large study [56]. However, it has been hypothesized that inflammation (i.e., higher C-reactive protein levels), which often coincides with the MetS, predicts PTSD symptoms in veterans [57], so there may be shared vulnerability underlying the link between PTSD and the MetS.

Future studies should replicate the findings of this study longitudinally and investigate the pathways in the link between mental health and the MetS. Because increased MetS risk is seen in several psychiatric patient populations, general mechanisms may link poor mental health to the MetS [58]. Our findings suggest that an increased risk of the MetS is not explained by an unhealthy lifestyle that may be a result of suffering from a mental disorder. However, we did not assess all lifestyle factors (e.g., nutrition and sedentary behavior) in much detail. Another pathway is biological stress dysregulation through disruption of the hypothalamic–pituitary–adrenal (HPA) axis [9, 59]. The disruption of the HPA axis has been linked to metabolic and cardiovascular disorders [60, 61]. Interestingly, persons with PTSD generally show lower cortisol levels, whereas in depressed persons these levels are generally higher [59, 62]. Another possible pathway is through inflammatory processes. Several studies show that persons suffering from PTSD or depression show higher level of inflammatory markers [8, 63], but this has also been observed preceding trauma and PTSD development [9]. Similarly, there is evidence that suggests shared vulnerability of depression and the MetS [6]. Evidently, the link between mental health and the MetS remains complex, with multiple pathways acting synergistically [58].

## Conclusions

In conclusion, this study shows a consistent association between a depressed mood and the MetS across ethnic groups. In contrast, the evidence for the association between PTSD symptoms and the MetS was only observed in the Dutch participants and not among ethnic minority groups, suggesting this association may be ethnicity dependent. When a depressed mood and severe PTSD symptoms were comorbid, the association with the MetS was not different. Future studies should replicate these findings and include more factors that may shed light on possible mechanisms underlying the link with the MetS.

## Electronic supplementary material

Below is the link to the electronic supplementary material.

Supplementary material 1 (DOCX 22 KB)
