# Supplementary material for: The association of depression and posttraumatic stress disorder with the metabolic syndrome in a multi-ethnic cohort: the HELIUS study
Source: Soc Psychiatry Psychiatr Epidemiol. 2018 May 23;53(9):921–30. doi: 10.1007/s00127-018-1533-y (PMC6133160; doi:10.1007/s00127-018-1533-y)
Supplement: Supplementary file 1 — Supplementary material 1 (DOCX 22 KB) [file 127_2018_1533_MOESM1_ESM.docx]

Electronic supplementary material

**Title:** The association of depression and posttraumatic stress disorder with the metabolic syndrome in a multi-ethnic cohort: the HELIUS study

**Journal:** Social Psychiatry and Psychiatric Epidemiology

**Authors:** Marieke J. van Leijden^1^, Brenda W.J.H. Penninx^2^, Charles O. Agyemang^1^, Miranda Olff^3^, Marcel C. Adriaanse^4^, Marieke B. Snijder^1^

**Affiliations:**

1: Department of Public Health, Academic Medical Center, Meibergdreef 15, 1105 AZ, Amsterdam, the Netherlands

2: Department of Psychiatry, Amsterdam Public Health Research Institute, VU University Medical Center, Oldenaller 1, 1081 HL, Amsterdam, The Netherlands

3: Department of Psychiatry, Academic Medical Center, Meibergdreef 5, 1105 AZ, Amsterdam, the Netherlands

4: Department of Health Sciences, Vrije Universiteit Amsterdam, De Boelelaan 1085, 1081 HV, Amsterdam, the Netherlands

**Corresponding author**

Marieke J. van Leijden: mariekevanleijden.amc@gmail.com

**Contents**

Table S1: Association (ORs with 95% CI) PTSD symptom categories with the MetS, in the total sample and by ethnicity

Table S2: : Associations (ORs with 95% CI) between PHQ-9 sum score (continuous) and the MetS, in the total sample and by ethnicity

Table S3: P- values interaction depressed mood (PHQ-9 sum score ≥10) X severe PTSD symptoms (PTSD sum score ≥7) in the association with the MetS, in the total sample and by ethnicity

**Table S1**: Association (ORs with 95% CI) PTSD symptom categories with the MetS, in the total sample and by ethnicity

|  | Total sample (*n*=21,182) | Dutch  (*n*=4527) | South-Asian Surinamese  (*n*=2999) | African Surinamese  (*n*=4058) | Ghanaian  (*n*=2251) | Turkish  (*n*=3522) | Moroccan  (*n*=3825) |
| --- | --- | --- | --- | --- | --- | --- | --- |
| Model 2 |  |  |  |  |  |  |  |
| No PTSD symptoms | 1 (ref) | 1 (ref) | 1 (ref) | 1 (ref) | 1 (ref) | 1 (ref) | 1 (ref) |
| Low (1-3 symptoms) | 1.08 (0.99-1.18) | 1.20 (0.98-1.47) | 1.16 (0.93-1.43) | 1.08 (0.90-1.30) | 1.08 (0.83-1.39) | 0.90 (0.73-1.11) | 1.12 (0.90-1.40) |
| Intermediate (4-6 symptoms) | 1.04 (0.93-1.16) | 1.08 (0.81-1.44) | 1.27 (0.97-1.66) | 1.10 (0.86-1.42) | 0.74 (0.51-1.10) | 1.01 (0.80-1.29) | 0.88 (0.68-1.16) |
| Severe (7-9 symptoms ) | 1.13 (0.99-1.28) | **2.06 (1.31-3.23)** | 1.02 (0.77-1.35)* | **1.40 (1.02-1.94)** | 1.19 (0.71-2.00) | 1.07 (0.84-1.36)* | 0.94 (0.72-1.23)* |
| Model 3 |  |  |  |  |  |  |  |
| No PTSD symptoms | 1 (ref) | 1 (ref) | 1 (ref) | 1 (ref) | 1 (ref) | 1 (ref) | 1 (ref) |
| Low (1-3 symptoms) | 1.06 (0.97-1.15) | 1.18 (0.96-1.45) | 1.12 (0.90-1.39) | 1.06 (0.88-1.28) | 1.06 (0.82-1.38) | 0.88 (0.72-1.08) | 1.09 (0.87-1.36) |
| Intermediate (4-6 symptoms) | 0.96 (0.85-1.07) | 1.01 (0.75-1.36) | 1.13 (0.86-1.49) | 0.99 (0.77-1.29) | 0.73 (0.49-1.08) | 0.95 (0.74-1.22) | 0.81 (0.61-1.07) |
| Severe (7-9 symptoms ) | 0.94 (0.82-1.08) | **1.77 (1.10-2.86)** | 0.79 (0.57-1.09)* | 1.08 (0.76-1.53) | 1.12 (0.65-1.95) | 0.94 (0.72-1.23)* | 0.79 (0.59-1.05)* |

Model 1: adjusted for age and sex (and for ethnicity in the total sample only)
Model 2: adjusted for age, sex and educational level (and for ethnicity in the total sample only)
Model 3: adjusted for age, sex, educational level and comorbid depressed mood or severe PTSD symptoms (and for ethnicity in the total sample only)
*p* value <0.05 are in bold
* Association is significantly different compared to the association in the Dutch population (*p* value interaction <0.05)

**Table S2**: Associations (ORs with 95% CI) between PHQ-9 sum score (continuous) and the MetS, in the total sample and by ethnicity

|  | Total sample (*n*=21,182) | Dutch  (*n*=4527) | South-Asian Surinamese  (*n*=2999) | African Surinamese  (*n*=4058) | Ghanaian  (*n*=2251) | Turkish  (*n*=3522) | Moroccan  (*n*=3825) |
| --- | --- | --- | --- | --- | --- | --- | --- |
| Model 1 | **1.03 (1.02-1.03)** | **1.05 (1.03-1.08)** | **1.03 (1.01-1.04)** | **1.04 (1.03-1.06)** | 1.01 (0.98-1.03)* | **1.02 (1.01-1.03)*** | **1.02 (1.01-1.03)*** |
| Model 2 | **1.02 (1.02-1.03)** | **1.04 (1.02-1.06)** | **1.03 (1.01-1.04)** | **1.04 (1.02-1.06)** | 1.01 (0.98-1.03) | **1.01 (1.00-1.03)*** | **1.02 (1.01-1.03)** |
| Model 3 | **1.03 (1.02-1.03)** | **1.04 (1.01-1.06)** | **1.04 (1.02-1.05)** | **1.04 (1.02-1.06)** | 1.01 (0.98-1.03) | **1.02 (1.00-1.03)*** | **1.03 (1.01-1.04)** |

Model 1: adjusted for age and sex (and for ethnicity in the total sample only)
Model 2: adjusted for age, sex and educational level (and for ethnicity in the total sample only)
Model 3: adjusted for age, sex, educational level and comorbid depressed mood or severe PTSD symptoms (and for ethnicity in the total sample only)
*p* value <0.05 are in bold
* Association is significantly different compared to the association in the Dutch population (*p* value interaction <0.05)

**Table S3**: *p* values interaction depressed mood (PHQ-9 sum score ≥10) X severe PTSD symptoms (PTSD sum score ≥7) in the association with the MetS, in the total sample and by ethnicity

|  | Total sample (*n*=21,182) | Dutch  (*n*=4527) | South-Asian Surinamese  (*n*=2999) | African Surinamese  (*n*=4058) | Ghanaian  (*n*=2251) | Turkish  (*n*=3522) | Moroccan  (*n*=3825) |
| --- | --- | --- | --- | --- | --- | --- | --- |
| Model 1 | 0.97 | 0.61 | 0.78 | 0.89 | 0.73 | 0.35 | 0.82 |
| Model 2 | 0.78 | 0.80 | 0.82 | 0.92 | 0.75 | 0.27 | 0.72 |

Model 1: adjusted for age and sex (and for ethnicity in the total sample only)
Model 2: adjusted for age, sex and educational level (and for ethnicity in the total sample only)
*p* value <0.05 are in bold
